# A national serosurvey to determine the prevalence of paratuberculosis in cattle in Bhutan following detection of clinical cases

**DOI:** 10.1002/vms3.114

**Published:** 2018-07-05

**Authors:** Ratna B. Gurung, Douglas J. Begg, Richard J. Whittington

**Affiliations:** ^1^ Department of Livestock National Centre for Animal Health Thimphu Bhutan; ^2^ Sydney School of Veterinary Science and School of Life and Environmental Sciences Faculty of Science University of Sydney Camden New South Wales Australia

**Keywords:** paratuberculosis, cattle, serosurveillance, prevalence, Bhutan

## Abstract

Johne's disease is an economically important ruminant disease predominantly affecting cattle, sheep and goats. The economic losses are due to early culling, reduced growth rate, progressive weight loss and reduced production. It is caused by *Mycobacterium avium* subspecies *paratuberculosis* (MAP). Johne's disease was reported in cattle in Bhutan, based on clinical signs and histopathology; in the late 1990s samples from one mithun that was suspected to have died due to this disease was confirmed by molecular testing at the Faculty of Veterinary Science, University of Sydney, Australia. However, no detailed study on prevalence of JD has been attempted in Bhutan. Objective of this study was to conduct serosurveillance to determine the national prevalence of Johne's disease in cattle for the period 2013–2014 to provide the basis for planning a future control strategy. A national serosurvey was conducted wherein a two‐stage sampling procedure was used with 95% confidence and an error level of ±0.05. The sample size required for the survey was calculated using the software‐Survey Toolbox for Livestock Diseases, available as Epitools at http://www.ausvet.com.au. A total of 1123 serum samples were collected from an administrative structure of 52 villages, 40 sub‐districts and 15 districts. Serum samples were tested using commercially available antibody enzyme linked immunosorbent assay. Statistical analysis was performed using GraphPad Prism 5.0. Illustration such as maps was produced using QGIS version 2.18 ‘Las Palmas. The mean national apparent prevalence of Johne's disease was found to be 2.31 (26/1123) (95% CI: 0.80–4.50) with an estimated true prevalence was found to be 8.00 (95% CI: 2.00–17.00). Trongsa district had the highest prevalence (12.96) followed by Zhemgang (4.34), Lhuntse (4.25), Sarpang (3.89), Bumthang (3.60), Trashigang (2.67) and Haa (2.63). Prevalence for all other districts was 2.00 or below. Seropositive samples were reported from all over the country with varying levels of sero‐positivity. In the recent past many more cattle were imported from India to boost dairy production. Nevertheless, the wide distribution of seroreactive JD cattle all over the country is a concern for future control. Therefore, in future, a detailed study on the impact of cattle import with regard to disease incursion such as Johne's disease and other diseases should be undertaken.

## Introduction

Nestled in the Himalayas and sandwiched between China to the north and India to east, west and south, the Kingdom of Bhutan is a small landlocked country with an area of 38 394 km^2^ stretching approximately 160 km north‐south and 240 km east‐west. The geo‐location of the country is defined by 26° 40′–28° 20′ northing and 88° 45–92° 70′ easting. The current human population of the Bhutan is 783 534 (NSB, 2016). As a result of the remarkable variations in altitude in a small area, the country experiences diverse climatic conditions from wet sub‐tropical in the south and temperate to alpine in the north. About 70.46% of land is under natural forest cover (NSB, 2016). Bhutan is administratively divided into 20 districts, 205 sub‐districts and 4340 villages (ECB, 2008) and is essentially an agrarian country with about 57% of the population engaged in agriculture for their livelihood (DoE, 2014). Livestock farming forms an integral part of the agricultural system with about 62% of the households rearing livestock (NSB, 2013).

The traditional farming system involves the integration of crop production and livestock rearing. The majority of the livestock farming practices involve grazing in agricultural fields and in forests. Only a small proportion of livestock are reared under a stall‐feeding system. Livestock farmers in the high‐altitude areas practice a traditional transhumance migration system (downward in winter and upward in summer) in an effort to make feed and fodder available for their animals all year. The country's ruminant population consists of 298 916 head of cattle and about fifty thousand head of small ruminants (DoL, 2012). Among ruminants, yaks and mithuns (syn. gayal*, Bos frontalis*) are reared in a semi‐wild management system.

Johne's disease is an economically important ruminant disease predominantly affecting cattle, sheep and goats. It is prevalent in many countries around the world. The economic losses are due to early culling, reduced growth rate, progressive weight loss and reduced production. It is caused by *Mycobacterium avium* subspecies *paratuberculosis* (MAP). In developed countries with intensive dairy farming systems, the herd level prevalence of Johne's Disease (JD) was reported to be as high as 50% (Ott *et al*. 1999; Nielsen & Toft 2009). Countries neighbouring Bhutan have reported comparatively lower prevalences. In Nepal, JD prevalence in goats was reported to be as high as 14.2% (Singh *et al*. 2007). The national prevalence in India is unknown but regional prevalences in different ruminant species are available. In north India which shares a border with Bhutan the prevalence of JD in cattle was estimated to be 28% (Singh *et al*. 2008). In south India, the prevalence of JD in buffalo was estimated at 21.3% (Sivakumar *et al*. 2005). In organized cattle and buffalo farms in Gujarat, one of the Indian states in the western region, the seroprevalences of JD were 6.8% and 5.8%, respectively (Mohan *et al*. 2009). These data are of concern because India has been the main source of Bhutan's cattle imports. In the past, high producing dairy stock were imported from various states of India, the southern neighbouring country either as replacement stock for government breeding farms or for distribution to farmers under government subsidy. In the absence of stringent disease screening regulations for imported cattle, disease incursions with imported live animals occurred. In addition to the importation of live ruminants, Bhutan also imports substantial quantities of dairy products from India for domestic consumption.

The zoonotic potential of Johne's disease has been debated for more than a century since it was first reported to be analogous to Crohn's disease clinically as well as pathologically (Grant 2005; Behr & Collins 2010). In the last three decades after the first isolation of MAP from a Crohn's patient in 1989, several studies have generated evidence of an association between MAP and Crohn's disease (Chiodini 1989). A systematic review and meta‐analysis of the zoonotic potential of MAP by Waddell *et al*. (2015) showed positive and consistent associations between MAP and Crohn's disease. Potential sources of MAP exposure for humans include animal origin food such as hamburger meat (Mutharia *et al*. 2010), infant milk formula (Hruska *et al*. 2005), milk (Van Kruiningen *et al*. 2005), cheese (Maconi *et al*. 2010), meat (Sakamoto *et al*. 2005), breast milk (Naser *et al*. 2000), water (Pickup *et al*. 2005; Whittington *et al*. 2005) and the environment (Eisenberg *et al*. 2011). In India, high rates of human exposure to MAP have been reported (Singh *et al*. 2014a,b). Control of JD in livestock is the most effective means of reducing human exposure to MAP.

There have been isolated reports of JD in cattle in Bhutan, detected based on clinical signs and histopathology; in the late 1990s samples from one mithun that was suspected to have died due to JD was confirmed by molecular testing at the Faculty of Veterinary Science, University of Sydney, Australia to have paratuberculosis (unpublished data). However, no detailed study on prevalence of JD has been attempted in Bhutan. Without such information on the status of JD in Bhutan, the animal health programmes in the country cannot include a specific control strategy for JD. The aim of this study was to conduct serosurveillance to determine the national prevalence of JD in cattle for the period 2013–2014 to provide the basis for planning a future control strategy.

## Materials and methods

### National serosurveillance design

A national survey was designed to collect serum samples from all cattle rearing areas in the country. Sampled animals were from mixed breeds and husbandry practices. All the villages except for those that do not rear cattle or fall under the jurisdiction of major municipalities were included in the study design. Attributed to the pathogenesis of the disease, which has a long incubation period making seroconversion unlikely in young animals, and also due to farmers’ reservations about bleeding younger animals, calves below 6 months of age were excluded from the study. A two‐stage sampling procedure was used. With 95% confidence and an error level of ±0.05 the design was expected to detect 10% prevalence. Based on probability proportional to size (PPS) and expected prevalence of 10%, the sample size required for the survey was calculated using the software‐Survey Toolbox for Livestock Diseases, available as Epitools at http://www.ausvet.com.au. The variances used for within villages and between villages were 0.20 and 0.04, respectively (Cameron 1999).

Simple random sampling (SRS) was applied at the first stage giving every village the same chance of being selected and stratified at district level. Although the total number of animals in each district and subdistrict was known, the total number of animals in each village was not available and thus it was not possible to select household as well as animal. Therefore, systematic random sampling was performed at the second stage to sample every second animal in the entire village so as to obtain a self‐weighted sample.

### Sample collection and storage

**Figure 1:**
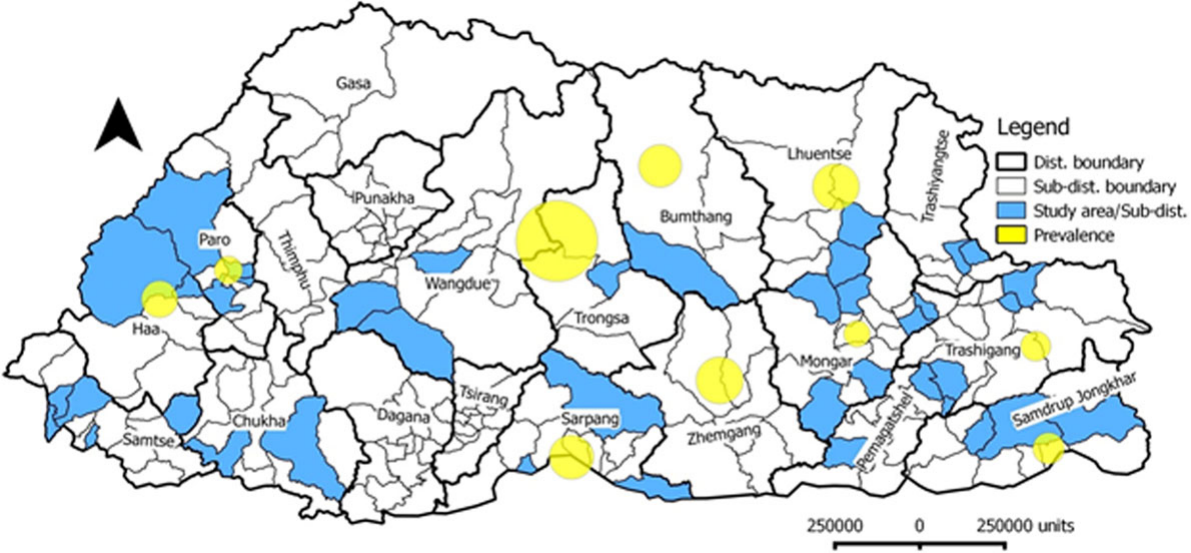
Map of Bhutan showing the study areas along with weighted prevalence of Bovine Johne's Disease in Bhutan.

A total of 1123 serum samples were collected from an administrative structure of 52 villages, 40 sub‐districts and 15 districts. Twenty two animals were sampled from each village. The sampling area distribution is shown in Figure 1. About 8 mL blood was collected from the jugular vein of all of the selected animals. Serum was separated from whole blood, stored at −20°C, transported to the National Veterinary Laboratory at the National Centre for Animal Health, Thimphu and archived at −20°C until tested.

### ELISA methodology

The IDEXX ELISA (Montpellier, France) was performed according to the manufacturer's instructions. Briefly, serum samples, including positive and negative controls, were diluted (1:20) in a buffer containing an extract of *Mycobacterium phlei (M. phlei)*, mixed with a shaker and incubated for 1 h at room temperature (RT). The adsorbed serum samples (100 *μ*L) were dispensed to each well of an ELISA plate pre‐coated with antigen, mixed with gentle tapping on the sides and incubated for 45 min at RT. The plates were hand‐washed three times with wash buffer, and a peroxidase‐labelled monoclonal anti‐ruminant IgG conjugate (100 *μ*L) diluted in dilution buffer (1:100) was added to each well and incubated for 30 min at RT. The plates were washed as above and 100 *μ*L of 3′, 3′, 5′, 5′ ‐ tetra‐methyl‐benzidine (TMB) substrate was added and incubated for 10 min in dark. The chromogenic reaction was stopped by adding 100 *μ*L of stop solution, and the optical density (OD) values were read at 450 nm using a plate reader (Wellkang Ltd., London, UK). The ELISA results were interpreted as the signal of the test sample as a proportion of the positive control, corrected for the negative control (sample‐to‐positive; SP%), according to the formula: SP% = [(OD_450 nm_ of Test sera − OD_450 nm_ of Negative control) ÷ (OD_450 nm_ of Positive control − OD_450 nm_ of Negative control)] × 100. SP% cut point for test sera ≥55% were considered positive, according to the manufacturer's instructions. Test sera with an SP% >45% and <55% were considered as suspect and re‐tested. The re‐test values were used for analysis. Test sera with an SP% ≤45% were considered negative.

### Analysis

Sample frame calculation was performed using Epitools (http://hub.epitools.ausvet.com.au). Maps for spatial distribution of prevalence and sampling sites were illustrated using QGIS version 2.18 “Las Palmas” (http://hub.qgis.org/projects/quantum-gis). Statistical analysis was performed using GraphPad Prism 5.0 (GraphPad Software Inc., La Jolla, USA). Apparent prevalence and the ranges (95% confidence limits) were calculated using EpiTools epidemiological calculator (AusVet) (Brown *et al*. 2001). Apparent prevalence percentage (AP, the proportion of cattle that are test positive) was used to estimate the true prevalence percentage (TP, the proportion of cattle truly infected with MAP) using the formulae TP = (AP + specificity − 1)/(sensitivity + specificity − 1) (Thrusfield 1995). The sensitivity and specificity of the test to calculate TP were 0.56 and 0.99, respectively. Confidence limits for TP were calculated by using the upper and lower 95% confidence limit estimates for AP in the above formula (Thrusfield 1995). Diagnostic estimates of the assay were used as per the manufacturer claim to calculate prevalence (IDEXX, 2015).

## Results

### Seroprevalence

The mean national apparent prevalence of JD in Bhutan was found to be 2.31 (26/1123) (95% CI: 0.80–4.50). However, the estimated true prevalence was found to be 8.00 (95% CI: 2.00–17.00). Prevalence rates in different districts of the country are presented in Table 1 and Fig. 1. Trongsa district had the highest prevalence (12.96) followed by Zhemgang (4.34), Lhuntse (4.25), Sarpang (3.89), Bumthang (3.60), Trashigang (2.67) and Haa (2.63). Prevalence for all other districts was 2.00 or below. The data stratified by district are presented in Table 1. None of the samples from five districts tested positive: Chukha, Pemagatshel, Samtse, Trashiyangtse and Wangdue. Districts in the east central region of the country appeared to have higher prevalence compared with other districts and region. All four districts (Bumthang, Trongsa, Zhemgang and Sarpang) of east central region showed higher prevalence (Table 1).

**Table 1:** District level point prevalence percentage with 95% exact binomial confidence limits and the approximated true prevalence

District	Apparent prevalence (%)		True prevalence (%)		Sub‐district	Village
	Point estimate	95% CL	Point estimate	95% CL		
Bumthang	3.60 (1/28)	0.60–17.70	6.40	0–30.00	Chumey	Urook
Chukha	0 (0/54)	0–6.60	NA	NA	Bongo	Pakshikha
					Phuentsholing	Changaykha B
Haa	2.63 (1/38)	0.50–13.50	4.00	1.00–23.00	Katsho	Bali/Junidra
					Bji	Yangthang
Lhuntse	4.25 (4/94)	1.70–10.40	5.00	1.00–17.00	Menji	Zham
					Tshenkhar	Goney
						Godong
						Changmadung
Mongar	1.38 (3/216)	0.50–4.00	1.80	1.00–16.00	Dremetse	Bikhar Thramlo
					Gongdue	Gorthongla
					Kengkhar	Shingchongri
					Narang	Raynangkhar
						Gonmchuzur
					Tshakaling	Zangtung
						Wadong
					Tsamang	Ganglapong
						Tokari/Artang
						Tokari/Leochung
Paro	1.58 (2/126)	0.40–5.60	2.00	1.00–8.00	Dopshari	Jangsa
						Kempa Phatom
						Kempa Phenshing
					Hungrel	Chhubjakha
					Lungnyi	Dzongdra Dramalo
					Tsento	Satsham
Pemagatshel	0 (0/92)	0–4.00	NA	NA	Decheling	Dhungphu gonpa
					Nanong	Denphu
						Raling
Samdrup Jongkhar	1.96 (1/51)	0.30–10.30	2.00	1.00–17,00	Martshala	Dengshingzor
					Serthi	Tangngakpa
					Wangphu	Shokshi
Samtse	0 (0/98)	0–3.80	NA	NA	Biru	Nigurey
					Denchukha	Beteni
					Yoeseltse	Ghalley goan
					Lahireni	Malbasey
					Sipsu	Peljorling B
Sarpang	3.89 (3/77)	1.03–10.80	5.00	1.00–18.00	Jigmechholing	Chungsing
					Sompangkha	Kencholing
					Umling	Dangling
Trashigang	2.67 (3/112)	0.90–7.60	4.00	0.00–12.00	Lumang	Khesing
						Kharphu
					Phongme	Shingringmo
					Radhi	Radi Pangthang
Trashiyangtse	0 (0/38)	0–0.9.20	NA	NA	Khamdang	Lengkhar
					Toetsho	Khene
Trongsa	12.96 (7/54)	6.40–24.40	22.00	10.00–43.00	Drakten	Samchoeling
Wandue	0 (0/22)	0–14.90	NA	NA	Bjena	Tarog
						Khelakhar
					Daga	Tsara
					Gasatshoom	Bjikha
Zhemgang	4.34 (1/23)	0.80–21.00	5.00	0.00–36.00	Nangkhor	Goling
Overall	2.31 (26/1123)	0.80–4.50	8.00	2.00–17.00		

CL, confidence limits; NA, not applicable.

## Discussion

In the present study, the highest sero‐prevalence was found in Trongsa district (12.96%) which can be considered low when compared with the 28% seroprevalence from north India (Singh *et al*. 2008). Apart from 50% herd level prevalence in European countries (Ott *et al*. 1999; Nielsen & Toft 2009) other studies have shown a varying range of animal level sero‐prevalence: as high as 19% in Austria (Dreier *et al*. 2006); 15% in Lower Saxony, Germany (Bottcher 1997); 2.4% in Italy (Lillini *et al*. 2005) and 4.4% in Germany (Donat *et al*. 2005). Considering the highest prevalence of bovine JD in Trongsa and potential source for spread to other areas, a strategic control plan may be essential. The distribution of prevalence of BJD in Bhutan did not indicate any specific pattern in terms of proximity to neighbouring Indian states or the interior parts of the country. Seropositive samples were reported from all over the country with varying levels of sero‐positivity. Some districts were recorded with very low seroprevalence. There were records from the past claiming that Trongsa district purchased several dairy animals from other districts within the country as well as from some of the north‐eastern states of India. However, due to the lack of adequate information, complete analysis on the association between the purchase or animal trade and high sero‐positivity could not be confirmed. In the recent past (2014–2016), many more cattle were imported from India to boost dairy production. Therefore, in future, a detailed study on the impact of cattle import with regard to disease incursion such as JD and other diseases should be undertaken. Nevertheless, the wide distribution of seroreactive JD cattle all over the country is a concern for future control.

Although diagnostic specificity of JD ELISA is very high, the sensitivity is still low. This has been demonstrated by several studies (Dubash *et al*. 1995; Hope *et al*. 2000; Gurung *et al*. 2015). Additionally, JD is one of the few bacterial infections that have association of active infection and shedding with high serum antibody titre (Collins *et al*. 2005; Begg *et al*. 2010). Therefore, in this study also, the ELISA test may have detected antibody in animals that had active infection and possibly shedding but missed those animals with subclinical infection.

As Bhutan depends on importation of livestock and livestock products from India where JD is prevalent in cattle, importation of large volumes of dairy and meat products may act as potential sources of MAP exposure for humans as reported by other studies in other parts of the world (Waddell *et al*. 2015). There is obvious risk of spreading MAP in ruminant population and possibly also to humans. To date, in Bhutan there is lack of information on Crohn's disease, but it is wide spread in many countries including India. There is a need to investigate Crohn's disease in Bhutan too. Studies have shown that there is a direct relationship between the magnitude of ELISA results and the odds of a cow shedding MAP in its faeces or milk (Collins *et al*. 2005); the detection of seropositive animals means that there is likely to be faecal shedding and shedding of MAP in milk. Therefore, the operation of dairy processing units with proper hygiene and pasteurisation technology is essential to prevent MAP exposure of humans in Bhutan.

The field part of this study was conducted with limited resources and the sample collection was conducted during routine veterinary visits, thus, it was not possible to collect detailed information on potential risk factors for JD. Therefore, in future, a study that will consider details such as live animal import, sex, age, breed and husbandry practices will provide more information on JD prevalence and its associated parameters. This is the first study of bovine Johne's disease conducted in Bhutan. The findings from this study will form basis for developing strategic control plan in future.

## Conflicts of interest

The authors declare that there is no conflict of interest.
